# Some Points in the Natural History of Caries of the Teeth, and the Value of Fillings for Its Arrest

**Published:** 1880-11

**Authors:** G. V. Black

**Affiliations:** Jacksonville, Ill.


AMERICAN JOURNAL

OF

DENTAL SCIENCE.

Vol. XIV. THIRD SERIES—NOVEMBER, 1880. No. 7.

### ARTICLE I.

### *Some Points in the Natural History of Caries of the Teeth, and the Value of Fillings for its Arrest.*

BY G. V. BLACK, D. D. S., OF JACKSONVILLE, ILL.

Perhaps the best way to estimate the value of fillings, is to study the results in connection with an extended study of the habits and tendencies of caries of the teeth when allowed to pursue its course. The best results in the study of medicine within the last half century, have been obtained by the close observations made on disease, which has been allowed to pursue its course without therapeutic interference. By this means many errors of opinion respecting the tendency of individual diseases have been corrected, and a better knowledge of them arrived at. Indeed it is now recognized that in order to know a disease well, it must be studied without therapeutic interference—by no other means can we so certainly arrive at its true nature, habits and tendencies. Having thus gained an understanding of the disease, we are ready to note understandingly the modifying influence of drugs—not before—for certainly we cannot be supposed to understand what good or evil a drug has done in changing the course of a disease, if we have no real knowledge of the course of the disease when not modified by drugs.

Each disease has its habits and tendencies, which are more or less fixed, and each case pursues a course very similar to each other case of like nature or type. Each specific disease also arises from a specific cause; always from that one cause, and from no other, as certainly as an oak arises from an acorn, or a bird from an egg. In this catalogue of specific diseases, we, of course, do not include those accidental diseases which run no specific course, and probably arise from causes purely accidental. Yet it is well understood that the progress of discovery is continually transferring important lesions to the list of specific diseases.

Decay of the teeth is certainly a specific disease, running a specific course, and evidently arises from a certain specific cause, but this cause is not as yet certainly known. It may be regarded as a malignant disease, in that its general tendency is to the entire destruction of the organ attacked. This malignancy, however, receives a decided check in the fact that there is absolute immunity for all the other organs of the body than the teeth, hence the disease is always strictly localized. Whether its specific cause be local or general, is not yet certainly known. Not being able to trace the cause, our only knowledge of the disease is its phenomena, and it is to these phenomema that we now wish to direct attention. In this paper we can only glance at a few points. It was no part of the plan of this paper to enter into a discussion of the *cause* of decay, but I may be pardoned for introducing a few propositions on that subject, which will in some measure represent my conception of the facts now known to me.

1. There is no decay without an acid condition of the fluids of the mouth.

2. If acidity alone be the cause of decay it must be due to a state or condition of the acid not yet known or recognized.

3. While an acid is not only always present, but is probably a necessity to the inception and progress of decay, there may be an agent acting in conjunction with the acid that is not yet known or recognized.

4. While there is no decay without the presence of an acid, there is not necessarily decay because of the presence of an acid.

5. Decay is in no wise dependent upon the physiological perfection or imperfection of tooth structure any farther than that imperfections of the surface of a tooth may afford favorable points for the beginnings of decay.

6. The force of vitality has no influence in the prevention of decay. But in living dentine decay progresses less rapidly, probably because the tubules being perfectly filled with the fibrils, tends to prevent the ingress of the materies morbi, and in this manner limits the surface upon which it may exert its influence, and more rapidly in soft teeth than in hard ones, because there is less basic substance to be broken down.

My object in experimenting with acids in motion, of which a partial report was made to the American Dental Association, also to the Mississippi Valley Dental Association, was to see if this change in the condition of the acid would produce a change in its action. My observations had led me to the conclusion that certain changes would be produced. The result of experiments, while they demonstrated a change of action, showed a different change from that anticipated by me before beginning. All my efforts to detect a special agent acting jointly with an acid for the production of decay, have so far entirely failed. I have never been able to satisfy myself, either by experiment, observation, or otherwise, that *Leptothrix Buccalis* or other fungi demonstrable with the microscope acted as such morbific agent, or stood in the relation of causation to decay of the teeth.

We will now leave the causes of decay entirely, and in the rest of this paper we will regard it simply as the result of a force, without reference to the cause or origin of this force.

Decay is disintegration of the tooth substance, molecule by molecule. It always begins on the surface of the tooth and slowly makes its way toward the central parts, spreading as it proceeds. The dentine is destroyed more rapidly than the enamel so that in most instances the dentine is burrowed out beneath the enamel in such manner that the diameter of the cavity just within is larger than the opening.

Decay has pretty definitely fixed habits as to the point upon a tooth that is attacked, and one of the most notable characteristics is that it never attacks a tooth upon a surface that is smooth and is constantly worn or is kept clean by the attrition of mastication, or the friction of the lips, cheeks or tongue. All such points are absolutely exempt from attack.

The points of attack may be divided into four classes:

1. Pits and grooves in the enamel.
2. Approximal surfaces.
3. Points which are from any cause habitually unclean.
4. At the junction of the cementum and enamel.

The *first class* is usually the first to make its appearance. It occurs in the pits and grooves of the enamel wherever found. In the molars, in the pits and grooves of the grinding surface, and in the groove which is often present in the duccal surface. In the bicuspids, in the pits and grooves of the grinding surface. In the incisors, in the pits often present in the lingual surface. In pits and grooves, the result of faulty formation in any of the teeth.

The *second class* occurs in the approximal surfaces of any or all of the teeth below the junction of the cementum with the enamel, and is usually the second to make its appearance.

The *third class* rarely makes its appearance except in uncleanly mouths or among those who habitually sleep with the mouth open. Their position is the smooth surfaces—usually the buccal or labial—of any or all of the teeth below the junction of the cementum with the enamel.

The *fourth class* is always accompanied with diseased gums, which has usually caused more or less exposure of the cementum and has its beginning just at the joining of the cementum and enamel. It is most generally seen in middle life or in old age. Very rarely in children or young persons.

The first two classes belong to youth or early maturity. If they are not seen until later it is on account of their slow progress. After watching this point critically, I have become satisfied that very few decays in these positions originate after the age of twenty-five years; and as these comprise about ninety per cent. of all decay, we may conclude that caries of the teeth, in its inception at least, is a disease of youth.

We wish now to call especial attention to two points. We all know that mouths are frequently met with in which there are many decays, which at middle life have not progressed so far as to do material injury. Many points have been attacked, but the destructive process has been extremely slow. Again we often find dentures in which very few decays have started, but these few have progressed with great rapidity, and several teeth have been lost, while perhaps all, or nearly all, of the remaining ones are free from decay. There are the following variations:

1. Cases in which *few* decays start and the destructive process is *slow*.

2. Cases in which *many* decays start and the destructive process is *slow*.

3. Cases occur where few decays start and the destructive process is rapid.

4. Cases occur where many decays start and the destructive process is rapid.

To represent these characteristics, we propose the terms "vis inita," beginning-power—from *vis*, power, force; and *ineo*, to begin—and "vis deleta," destroying-power—from

*deleco*, to blot out, to destroy. With these words representing the quality of the power of caries, we may associate numbers to represent our conception of the degree of that power in a given case.

The following are the extremes of possible combinations:

Vis inita 1, with vis deleta 1 to 100.

Vis inita 100, with vis deleta 1 to 100.

For some years past we have made a special study of some of these characteristics of decay, particularly those of which we now speak, and we are satisfied that a more close and thoughtful study of them will be of very great benefit, both to operator and patient, and at the same time serve to correct many misapprehensions as to the real value of fillings. And we have thought that the selection of some simple words not in common use, which might be clothed with a specific meaning to represent them, would not only be acceptable but helpful.

Now, as to the character of these powers, we do not for a moment wish to be understood as attributing the beginning of decay, the vis inita, and the destructive power, vis deleta, to different causes, but should rather say that these are manifestations of one force, but of different qualities of that force, or manifestations under differences of environment. In the one case the action is on the surface of the tooth, or in a shallow pit or groove, where the general fluids of the mouth have tolerably free access to the part or tissue being acted upon, while the other is much more secluded from disturbing influences—is deeper in the tooth—more hidden away. Again, the vis inita is exerted for the most part upon enamel, while the vis deleta is spent mostly upon the dentine, or with our present lack of knowledge of the cause of decay, it might be regarded as an arbitrary division for the study of phenomena. Whatever may be the cause of the differences, it is clear enough that they exist and modify powerfully the conditions under which we are called upon for dental operations, and exert a great influence upon the value or protective power of fillings.

It is not uncommon to find persons who have had no dental operations whatever, with some teeth missing, or only roots remaining here and there through the mouth. An examination proves that most of the other teeth are free from decay. The teeth that are wanting have been attacked and quickly destroyed by decay, while the remaining ones have not been attacked at all. In this case we would say, perhaps, that there was vis inita, 25; vis deleta, 90.

In another case we may find the denture of a patient at middle life with many small, dark cavities, none of which have progressed so far as to give any trouble. For the practical purposes of mastication the teeth are as yet uninjured. Only a short time ago I examined such a case, in which I should say there was vis inita, 80; vis deleta, 10. Many decays had started in the teeth, but that power which drives on to quick and sure destruction, which we so often see to our sorrow, was wanting.

It was very different with a pair of twins, 18 years old, Swedish girls, who came to me for advice, last October. In the mouths of these girls there was not a tooth, except the wisdom teeth (which were not yet through the gums,) that were not attacked at from one to four points; not a tooth which was not from one-half to three-fourths destroyed by caries; in nearly every tooth there was a living exposed pulp. This, as being the most complete case of destruction I have ever met with, I should place vis inita, 100; vis deleta, 100, as the proper expression of the activity of the force there in operation.

We need not continue the recitation of these cases farther at present, as we think our meaning will be readily understood; but will now turn our attention to the modifying influence of age, or the different expressions of these powers usually found at different periods of life.

Decay of the teeth is essentially a disease of youth. Especially is this the case with the first and second classes of decay—those starting in the pits and grooves and on the approximal surfaces of the teeth, and to a somewhat lesser extent with the third class. With these, the first and second classes, the vis inita is most strongly manifested before the age of twenty years, and grows less and less active after that age; and perhaps ceases altogether as early as the fortieth year, except in occasional cases. As the first two classes include most of the cavities met with, we find that if the decays occurring up to this time are successfully protected by fillings, the work of the dentist is about accomplished.

With the vis deleta the case is very different—this may continue for a much longer period. It is less modified by age. We well remember a case which came under our care fifteen years ago. We all remember such cases. The patient was a maiden lady, twenty-eight years old. The vis inita had been very strong as was evident from the fact that almost every tooth remaining in the mouth—five had been lost—had been attacked at from one to three points, except the lower incisors. The vis deleta was less strong, say fifty, but by this time had made sad havoc, as the previous attempts at filling had not been very effectual. The patient wished to have the teeth out and try artificial ones. We protested and made fillings instead. Since that time no new cavity has occurred. Eight years afterwards she was married and has borne one child. During this pregnancy two of the cavities, the fillings in which had been observed to be slightly leaky at the margins, flamed out into rapid decay (vis deleta, strong.) These points were attended to and no decay has occurred to this day. (These were not plastic fillings.) In this case the vis inita had become almost or quite nothing, with possibly a diminishing of the destructive force, and the remaining vis deleta was mastered by reasonably good fillings until an increase of this force, during pregnancy, searched out and exposed the weak points. Three younger sisters of this patient came under my care at the same time. For them I made many fillings, but no tooth has been lost, and for the last five years no filling has been needed.

We might cite very many cases to show that the beginning power of decay is exerted mostly in youth, but deem it unnecessary. The time at which teeth are attacked varies very much in different cases, depending seemingly upon three conditions.

The first is the vis inita, second the physical perfection of the surface of the enamel; third, habitual cleanliness. It will be readily seen that teeth with deep pits and fissures will be attacked in the presence of a weak or moderate vis inita, when teeth of greater physical perfection would escape attack altogether. The same is true of teeth habitually unclean as compared with those that are clean. Both of these conditions vary the manifestations of the vis inita powerfully, favoring it in the one case and disfavoring it in the other. Therefore we have these three considerations before us in the study of the beginnings of decay. If the vis inita is naught we will have no decay. We must have a power or force acting to initiate the process. Mere pits, grooves, or other faulty formation, nor even uncleanness will induce it, unless, indeed, uncleanness carries with it the materies morbi. While if the vis inita be present, these circumstances will favor or disfavor its manifestation, and if the teeth be unequal in surface perfection, or habitual cleanliness, the expression of the vis inita will be unequal.

As we have said, this force is manifested mostly in youth, and when it is strong the teeth are attacked usually at about a certain time after coming through the gums. That is, if the first molar is found with a decay in its grinding surface two years after coming through the gums, or at eight years, the second molar is likely to have a similar decay when about the same age, or when the patient is fourteen years old. If the incisors have approximal decays five years after coming through, the bicuspids and first molar are likely to have approximal cavities at about the same time after they present themselves, or possibly the first molar may be attacked while approximating with the second deciduous molar, and so on with the other teeth in their turn.

Thus we see why it is that we have new cavities occurring continually between the ages of ten and twenty especially, and sometimes continuing until later in life, when the vis inita is less pronounced or more fluctuating. Of course we only present this as the most general rule to which the exceptions are sufficiently numerous. The modifying influences of surface perfection and cleanliness have their weight, which is often very powerful, in modifying the results, and if these are unequal among the different teeth, the manifestations will be unequal. After these come changes of condition as to health with their modifications; conditions which we need not now discuss, as we have considered them at length in a former paper. We will also find that the vis inita often begins to diminish very early in life, so as to lengthen the time between the occurrence of cavities in the different teeth.

Running all through our cases, however, there are many exceptions and exemptions. For instance, the lower incisiors are exempt from decay in a very large majority of cases. This is so very decided that we not unfrequently find them sound when all the other teeth have been destroyed. Again, it may happen that some other class of teeth are especially exempt, as the superior incisiors, or the canines—occasionally the bicuspids—but very rarely the molars. The wisdom teeth, too, often decay much earlier proportionately, than the other teeth, probably from some peculiarities of environment. All this tends to cover up and conceal the laws of which we have spoken. But when we come to compare a large number of examinations, or better still, to follow a large number of patients from childhood to middle age, the general rule stands out clearly and unmistakably.

The vis deleta—destroying power—of caries is also subject to various local modifying influences. It is especially and powerfully modified by the condition of the cavity as to its opening, progressing far more rapidly if the opening be small than if it be large, so as to give free ingress and egress to the fluids of the mouth. Indeed if the vis deleta be rather weak, decay, which is moderately progressive in a cavity, the opening of which is small, or partially closed, will cease altogether, if it be thrown wide open to the fluids of the mouth. Just here there is a very curious phenomena, which I have studied with much interest. Decay seems to be like the miner cutting coal. To work to advantage he must have a certain amount of room in which to handle his pick. If you cramp him he will go very slowly until he gains room for himself, then he will proceed rapidly with his work. It is just so with decay. While it is true that to progress most rapidly, the opening of the cavity should be partially closed, a moderately close filling of the cavity will interfere with progress for a time, until elbow room—if I may use such an expression—is gained.

It is for this reason that leaky fillings seem to protect for a time, especially in those cases in which the vis deleta is rather weak. However, if the vis deleta be strong, say 75, elbow room (figuratively) is gained so quickly that the decay seems to go right on without check.

Many noticing this in cases of very mild vis deleta, have come to the very erroneous conclusion that it is not necessary that fillings be water tight.

The changes in the destructive power consequent upon the breaking away of the wall of the cavity, and thus throwing it wide open to the fluids of the mouth, and still better to the attrition of mastication, we have seen exemplified in many ways. I was particularly struck with this last summer in my examination of the old Indian remains on the Mississippi. I spent some weeks in the work, and the results developed some curious facts not before known to me. One of these was the greater prevalence of decay among them than I had previously supposed. The second was the peculiar character of the decay. In a large majority of cases it was confined to the second class, nothing found but approximal decays. A very large proportion of these progressed until the over-laying enamel gave way, and then the hard food wedging into this broke out one side thus exposing the cavity to the attrition of mastication, which, with them, was sufficient to keep it clean and the decay came to a full stop leaving the teeth practically uninjured.

I did, however, find some dentures that had been practically destroyed. One, especially, of a young woman—the wisdom teeth just coming through—in whom the teeth were nearly all destroyed. There were also evidences of very severe alveolar abscesses which had made extensive burrows in the bony tissues.

This Indian girl seems to have been especially unfortunate, as she had had one femur and both bones of the forearm broken. The femur had healed witht wo inches shortening, and the arm with about one inch, and quite crooked. I also found a child six years old, with the temporary teeth very badly decayed.

The peculiarities above mentioned, of decay among the Indians, are sometimes seen among our own people. Decays are only found on the approximal surfaces, and the vis deleta being very weak the cavities are broken into before exposure of the pulp takes place and the decay comes to a full stop. The teeth are thus preserved through these natural and unaided processes. Such cases have occasionally come under my observation, as they doubtless have of most dentists of long experience.

Decays arising from want of cleanliness vary greatly as to time of life, but I am convinced that they occur mostly in youth or early maturity. How many decays of the first and second classes are really due to this cause is, of course, very difficult to estimate. I therefore include in this class only those that occur on the smooth and exposed surfaces of the teeth. They are seen mostly on the labial surfaces of the incisors and the buccal surfaces of the bicuspids and molars below the margin of the gum, always below the junction of the cement with the enamel. I think that I have sufficiently demonstrated the dependence of these decays upon uncleanness, or a local modification of the mucus, in my repeated experiments. It is well known that in these cases the tendency is for tooth after tooth to be attacked in succession, spreading either way from the point of beginning, (of course I do not allude to decays starting in congenital or other pits in these surfaces.) I have uniformly found that the daily use of the friction of the tooth-brush would prevent more teeth being attacked in this way.

We have yet to notice the fourth class of decay—those that occur at the margin of the enamel, or at the junction of the cement and enamel. These are peculiar to middle life and advanced age, occurring but rarely in young persons. We believe that the immediate local conditions of their occurrence are to be found in disease of the gingival margins of the gums, and perhaps the margins of the alveolar processes. This is marked by a thickened and irritated condition of the gums at the point, and usually some shrinkage away from the tooth. Some years ago I was inclined to the opinion that this condition was caused by the sharp edges of the cavity, but longer and closer observation has fully convinced me that the disease of the gum is first present, and the decay follows. The decays are not confined to any particular side of the tooth, but occur on the approximal as well as on the buccal and labial surfaces. Such decays also occur frequently where a partial plate abuts against the teeth, causing continued irritation of the margin of the gums. They sometimes progress rapidly and are very destructive, tooth after tooth falling a prey to their ravages. It occasionally happens that after the danger from the first and second classes of decay has passed, this fourth class springs into existence and wrecks the whole denture. We have lost the battle in several of these cases; in others, we seem for the time to have won the fight; and we have now under our care, several cases in which we are several times per year filling decays of this class.

VALUE OF FILLINGS.

In considering the value of fillings, we must at all times keep clearly in view the nature and habits of decay; its phenomena and tendencies—its particular characteristics in each individual case. If we let this escape attention in any case presented, we will have nothing upon which to base just conclusions. For instance, if we should take a patient at middle age, with a number of small, dark cavities, one in whom the vis inita is spent and the vis deleta is, and always has been very weak, and fill these cavities with amalgam, they would, if reasonably well done, remain good, and we might be inclined to point to the case as a grand success.

Again, let us take another case: The patient, a girl of fifteen or seventeen years, with a number of rapidly destructive decays, say vis inita, 50; vis deleta, 75, and make the best possible gold fillings in all apparent cavities. Within a few years this girl will most certainly show a number of decays advancing, and unless the fillings are absolutely perfect, there will be points about some of them that will need repair, or even if they be perfect, new points of decay are liable to present themselves in the immediate neighborhood. Now, we might be inclined to regard this case as to some extent at least a failure. In comparing these two cases without considering the nature of the diseased action with which we had to contend, we would certainly conclude that the amalgam had been the more successful, and therefore the better material; when, in fact, the reverse is true. The protective power of the gold fillings has really been very much greater than that exerted by the amalgam fillings, as would be shown if the cases were reversed. In the first instance the vis inita having been spent, there was no inclination to the formation of new cavities, and the vis deleta having always been weak and growing weaker, would easily be controlled by fillings approximately good, while the other case required absolute perfection in the filling of the cavities apparent, with a certainty of the appearance of new cavities in the near future.

This study of the case in hand, with a clear understanding of the phenomena of decay in their several degrees of manifestation, is a necessity to any just conception of the protective power of fillings. A few years ago it was announced by some one that it was not necessary that fillings should be absolutely moisture tight. While this proposition is false both in principle and in fact, observations made without an understanding of the degrees of the manifestation of the forces of decay, or proper consideration of them might seem to support such a conclusion. Every dentist of long experience has seen fillings manifestly imperfect, leaky, with a brown streak passing into the cavity on one side which have stood so for years. There has been enough action to produce a stain—show the leak, but the vis deleta being very weak becomes like the aged and feeble *miner* wedged in between two solid walls with his pick, without proper room to wield it. He works away feebly for awhile but finally smothers and dies. If, however, the vis deleta be strong, it is more like the *miner* in the prime of his strength, who, though cramped in a narrow crevice, soon gains elbow room and then wields his pick vigorously. Leaky, imperfect fillings afford protection only when but little protection is needed. Perfect fillings will afford protection to their cavities' walls under any ordinary circumstances.

The requirements of a filling of the strongest protective power are:

1. That the material be capable of *perfect* adaptation to cavities' walls, and of retaining that adaptation indefinitely.

2. That it be sufficiently resistant to the fluids of the mouth.

3. That it be sufficiently resistant to attrition.

No filling material yet in use has any continued virtue by reason of its intrinsic qualities as a material other than these. It is only by virtue of adaptation to the cavities' walls and the exclusion of the materies morbi that protection is secured. Those who use any material with any other end in view, must certainly fail in the end, no matter how profound a study they may make of compatibilities with tooth-bone, dentos, or what not, and the further their material departs from the three essentials given above, the more signal will be their failure.

These conclusions are based upon the observations of the greatest number of the wisest members of the dental profession. Then we have had a tolerably large personal experience, and fortunately we have kept such records of our operations that we may know whereof we speak. Among these records the number of failures (we say it in sorrow) is ample for comparison with the success.

The very best way of studying the value of fillings is to run over the records of those children who came to us years ago, and who have grown up to manhood and womanhood, to middle age, under our personal care. In studying these we must also estimate the value of the hereditary tendency of caries, for we all know that the especial liability to caries is more or less transmissible, Therefore, the history of the decay of the teeth of the parents must be known and considered. How many children have come under my care within the seventeen years that I have been in my present location, I do not know. Many come for a time and are gone and lost sight of, so that the number who have been constantly under my care and under the care of no other, is small, as I suppose it will be found to be with others. We can count about thirty who came to us, at from eight to fifteen years old, and whose parents, one or both, had lost their teeth from decay. These patients are now from twenty to thirty-two years old, and not one of them, nor any other who has been regularly under my care from childhood, wears artificial teeth of any kind, or needs to wear them. Not a tooth has been lost that had not an exposed or dead pulp at the time we first saw them, except such as were extracted on account of irregularity, *and no plastic fillings have been used.*

No table I am able to construct will give a fair shewing of these cases. The vis inita differs very considerably, so that the decays have occurred at very different periods as to age, which would confuse any table we can devise. Perhaps I should, in justice to myself, say a word, before giving cases from my records, in regard to some of my habits of practice which probably differ somewhat from my fellow-practitioners. For instance, in case I conceive the vis inita to be strong, I never, or very rarely, make what is called a pit-filling in the grinding surface of either the molars or bicuspids, but when a filling is required in one pit, I cut away the other, and all radiating fissures, uniting them in one filling. In the approximal surfaces I cut away all the approximating surface whether the cavity be large or small. For these reasons the number of fillings is reduced, as I think, nearly, or quite one half from what it would be if I filled every little cavity and stained pit. We will now give a few cases illustrating the success that may be attained by fillings.

Miss F. came under my care in 1866; was at that time 14 years old. Her father had lost all but the incisors and cuspids, and these were each protected by fillings, both upper and lower. The mother had lost all but the incisors in the lower jaw and a part of the anterior teeth above.

| | | | | | | | | | | | | |
|---|---|---|---|---|---|---|---|---|---|---|---|---|
| Year | 1866; | age | 14; | 5 | decays of 1st | class; | 1 | of 2d | class; | Total, | 6 |
| " | 1867 | " | 15 | 3 | " | " | 1 | " | " | " | 10 |
| " | 1868 | " | 16 | 1 | " | " | 1 | " | " | " | 12 |
| " | 1871 | " | 19 | 1 | " | " | 2 | " | " | " | 15 |
| " | 1872 | " | 20 | 3 | " | " | 1 | " | " | " | 19 |
| " | 1873 | " | 21 | 1 | " | " | 0 | " | " | " | 20 |
| " | 1874 | " | 22 | 1 | " | " | 0 | " | " | " | 21 |
| " | 1875 | " | 23 | 0 | " | " | 2 | " | " | " | 23 |
| " | 1877 | " | 25 | 0 | " | " | 3 | " | " | " | 26 |
| " | 1878 | " | 26 | 0 | " | " | 2 | " | " | " | 28 |
| " | 1879 | " | 27 | 0 | " | " | 2 | " | " | " | 30 |
| " | 1880 | " | 28 | 2 | " | " | 1 | " | " | " | 33 |

There was one repair of a filling in 1867; 1 re-filling in 1871; 1 re-filling in 1872.

These re-fillings were in the first lower molars. In these the pulps were dead and alveolar abscess formed when the patient came under my care. The walls of the cavities broke away, and the crowns were cut down half way to the gums, and re-filled to that height, and they are doing duty in that shape. No other filling has been lost, no tooth has been lost, and there are now no imperfections worthy of notice apparent about the margins of any of the fillings. *No plastics were used.*

Miss S., aged 12 years. The mother wears full plates. The father had lost more than half of his teeth when he came under my care, and the rest were protected by about twenty excellent fillings.

| | | | | | | | | | | | | |
|---|---|---|---|---|---|---|---|---|---|---|---|---|
| Year | 1870; | age | 12; | 2 | fillings first | class; | 4 | second | class; | | Total, | 6 |
| " | 1873 | " | 15 | 4 | " | " | 2 | " | " | | " | 12 |
| " | 1874 | " | 16 | 0 | " | " | 2 | " | " | | " | 14 |
| " | 1877 | " | 19 | 4 | " | " | 6 | " | " | | " | 24 |
| " | 1878 | " | 18 | 0 | " | " | 2 | " | " | | " | 26 |
| " | 1879 | " | 19 | 1 | " | " | 1 | " | " | | " | 28 |
| " | 1880 | " | 20 | 0 | " | " | 4 | " | " | | " | 32 |

One re-filling in 1878; 1 re-filling in 1880.

The patient was married at 18 and has borne one child.

We give these cases from our record as fairly representing the average results in the thirty cases alluded to, and with these two, the whole number as representing the results that may be obtained with gold fillings, believing that no such success can be attained with any plastic material now known to the profession.

These cases though not the worst, are bad cases, as the number of fillings show, and all past experience proves that there would have been almost complete destruction of the teeth but for the fillings. The patients, however, have done their part of the work. They have obeyed instructions and have been prompt to report anything wrong, otherwise we believe such success unattainable with any material.

In several families under my care, a part of the children have been prompt while others have been neglectful. The difference in result is strikingly apparent, and we think demonstrates unmistakably that a larger number of decays will occur if cavities are allowed to progress unchecked.

My most signal failures have been in those cases that come to me with the teeth badly wrecked between the ages of 18 and 22, especially in women who marry young and bear children. In such cases I have learned to expect some fillings to fail and that new cavities will occur rapidly. A close watch must be had for several years in order to secure good results. It would seem as though with the unchecked riot of the carious process, an *intensity* is attained which requires an unusually great effort to check; and indeed it seems it cannot be checked at once, but must be brought about by a persistent effort. It may be compared to checking the movement of a ball that has acquired a great momentum.

Older patients with similar conditions as to the progress of the decay are easier to manage in proportion to the age (provided the decay be not of the fourth class,) that is, given a certain amount of destruction by decay; the case is easier managed if the patient be 30 than if he be 18 or 20 years old.

We will give a case illustrating this point: A young lady, 17 years old, came under my care in 1870; four teeth were extracted, and fillings were made as follows:

| | | | | | | | | | | |
|---|---|---|---|---|---|---|---|---|---|---|
| At 17, | 9 | of the | first | class; | 5 | of the | second | class.... | Total, | 14 |
| " 20, | 2 | " | " | " | 13 | " | " | " .... | " | 15 |
| " 23, | 2 | " | " | " | 0 | " | " | " .... | " | 2 |
| " 24, | 1 | " | " | " | 1 | " | " | " .... | " | 2 |
| " 25, | 0 | " | " | " | 3 | " | " | " .... | " | 3 |
| " 27, | 0 | " | " | " | 0 | " | " | " .... | " | 0 |
| Totals, | 14 | | | | 22 | | | | | 36 |

*One refilled in* 1877.

Now, this patient returned three years after the first fillings were made with fifteen new cavities, with her teeth again in very bad condition, yet every filling previously made was perfect. It may be that a number of these new cavities were in progress, but as yet very slight, when the first fillings were made; but at the time nothing more was thought to be required than close watch for new cavities, which it seems the patient did not do. After the filling of these cavities the case seemed to be fairly under control, and I now suppose that a filling once in three or four years will be all that will be required. *No plastics.*

Another case: A lady came under my care, aged 28, at about the same time. The condition of the mouth was rather worse than the preceding. Eighteen fillings were made at first. After that time two fillings and two repairs complete the list of operations.

These cases illustrate the general rule, but of course we meet with exceptions. I have gradually come to understand that my better class of patients, who came to me as children and are prompt to report at the first intimation of anything wrong, cease to be remunerative patients after the age of thirty, provided that nothing but gold fillings have been made.—*Trans. of Ill. State Dental Society.*

---

